# 2-Methyl­sulfonyl-1,2,4-triazolo[1,5-*a*]quinazolin-5(4*H*)-one

**DOI:** 10.1107/S1600536812021769

**Published:** 2012-05-19

**Authors:** Rashad Al-Salahi, Mohamed Marzouk, Mohammed Abbas, Seik Weng Ng

**Affiliations:** a Department of Pharmaceutical Chemistry, College of Pharmacy, King Saud University, Riyadh 11451, Saudi Arabia; b Department of Chemistry, University of Malaya, 50603 Kuala Lumpur, Malaysia; c Chemistry Department, Faculty of Science, King Abdulaziz University, PO Box 80203 Jeddah, Saudi Arabia

## Abstract

The triazoloquinazoline fused-ring system of the title compound, C_10_H_8_N_4_O_3_S, is essentially planar (r.m.s. deviation = 0.027 Å). In the crystal, adjacent mol­ecules are linked by N—H⋯O_sulfon­yl_ hydrogen bonds, generating a helical chain running along the *b* axis.

## Related literature

For the synthesis of the precursor, see: Al-Salahi & Geffken (2011 ▶).
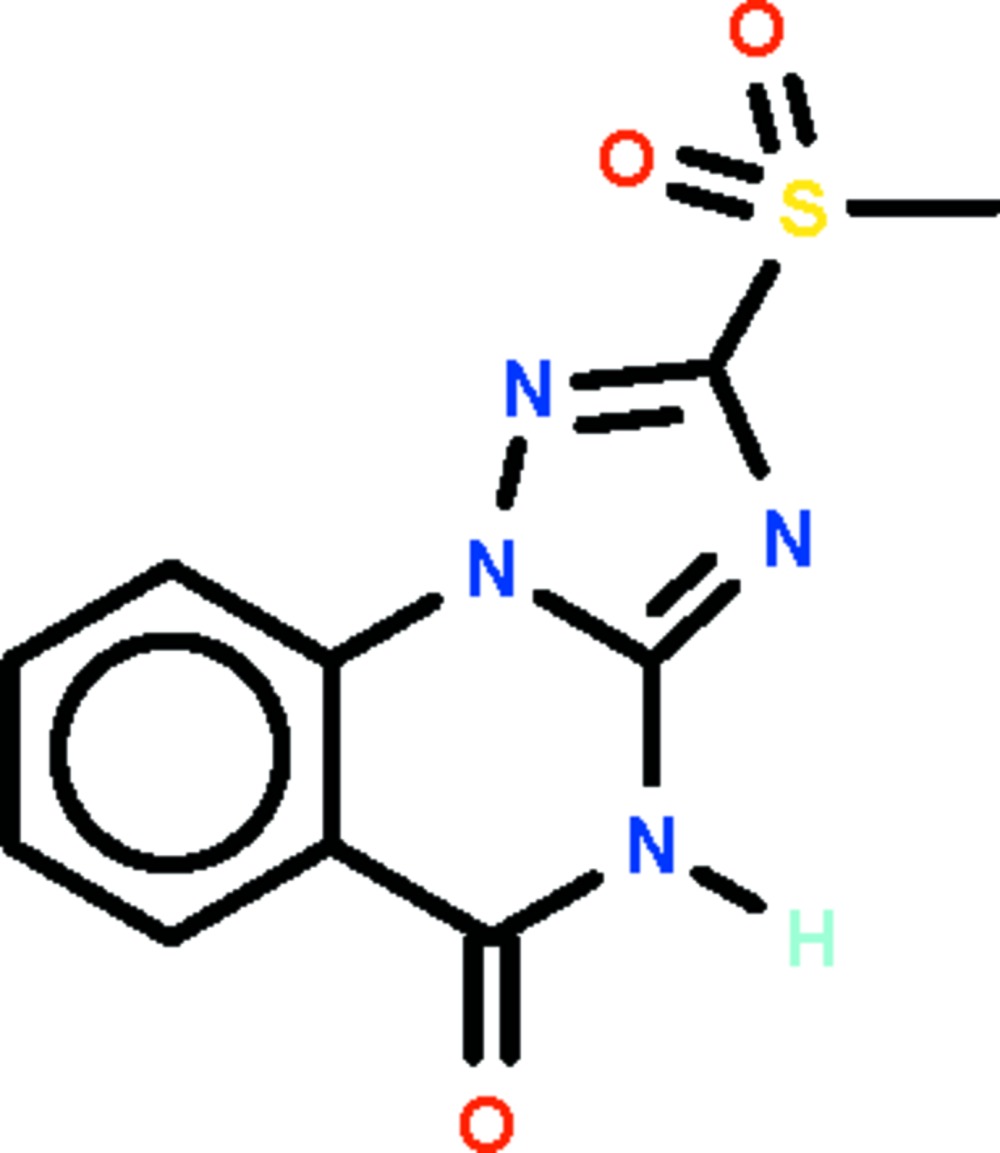


## Experimental

### 

#### Crystal data

C_10_H_8_N_4_O_3_S
*M* *_r_* = 264.26
Monoclinic, 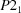

*a* = 9.6216 (2) Å
*b* = 4.9206 (1) Å
*c* = 12.1623 (3) Å
β = 106.628 (2)°
*V* = 551.73 (2) Å^3^
*Z* = 2
Cu *K*α radiation
μ = 2.71 mm^−1^
*T* = 294 K
0.20 × 0.10 × 0.02 mm

#### Data collection

Agilent SuperNova Dual diffractometer with an Atlas detector
Absorption correction: multi-scan (*CrysAlis PRO*; Agilent, 2012) ▶ *T*_min_ = 0.613, *T*_max_ = 0.948
9640 measured reflections
2304 independent reflections
2237 reflections with *I* > 2σ(*I*)
*R*_int_ = 0.025

#### Refinement

*R*[*F* ^2^ > 2σ(*F* ^2^)] = 0.030
*wR*(*F* ^2^) = 0.082
*S* = 1.04
2304 reflections
168 parameters
1 restraint
H atoms treated by a mixture of independent and constrained refinement
Δρ_max_ = 0.15 e Å^−3^
Δρ_min_ = −0.26 e Å^−3^
Absolute structure: Flack (1983 ▶), 1007 Friedel pairs
Flack parameter: 0.00 (2)

### 

Data collection: *CrysAlis PRO* (Agilent, 2012 ▶); cell refinement: *CrysAlis PRO*; data reduction: *CrysAlis PRO*; program(s) used to solve structure: *SHELXS97* (Sheldrick, 2008 ▶); program(s) used to refine structure: *SHELXL97* (Sheldrick, 2008 ▶); molecular graphics: *X-SEED* (Barbour, 2001 ▶); software used to prepare material for publication: *publCIF* (Westrip, 2010 ▶).

## Supplementary Material

Crystal structure: contains datablock(s) global, I. DOI: 10.1107/S1600536812021769/bt5915sup1.cif

Structure factors: contains datablock(s) I. DOI: 10.1107/S1600536812021769/bt5915Isup2.hkl

Supplementary material file. DOI: 10.1107/S1600536812021769/bt5915Isup3.cml

Additional supplementary materials:  crystallographic information; 3D view; checkCIF report

## Figures and Tables

**Table 1:** Hydrogen-bond geometry (Å, °)

*D*—H⋯*A*	*D*—H	H⋯*A*	*D*⋯*A*	*D*—H⋯*A*
N1—H1⋯O2^i^	0.88 (1)	2.23 (1)	3.072 (2)	160 (3)

Symmetry code: (i) 
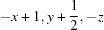
.

**Fig. 1.**
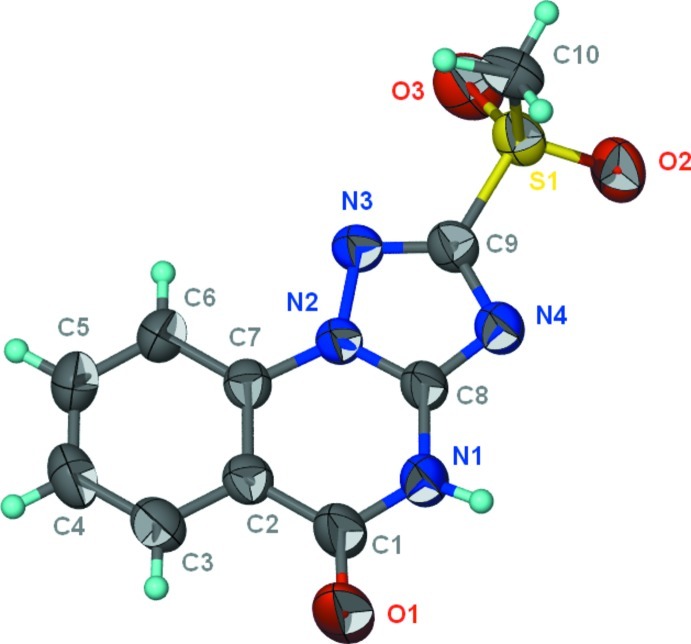
Anisotropic displacement ellipsoid plot (Barbour, 2001) of C10H8N4O3S at the 70% probability level; hydrogen atoms are drawn as spheres of arbitrary radius.

**Fig. 2.**
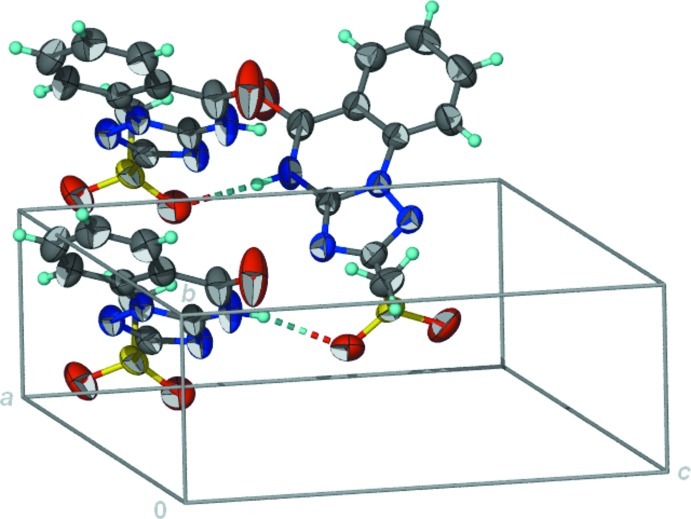
Hydrogen-bonded chain motif.

## supplementary crystallographic information

### Comment

2-(Methylsulfanyl)-[1,2,4]triazolo[1,5-*a*]quinazolin-5-one was synthesized from 2-hydrazinobenzoic acid and dimethyl *N*-cyanoimidodithiocarbonate; further reactions on the inherent lactam unit yielded other derivatives (Al-Salahi & Geffken, 2011). In the present study, this compound is oxided by hydrogen peroxide. The triazoloquinazoline fused-ring system of C_10_H_8_N_4_O_3_S (Scheme I, Fig. 1) is planar. Adjacent molecules are linked by an *N*–*H*···O_sulfonyl_ hydrogen bond to generate a helical chain running along the *b*-axis of the monoclinic unit cell (Fig. 2, Table 1).

### Experimental

Under ice-cold conditions, 2-hydrazinobenzoic acid (10 mmol, 1.52 g) was added to a solution of dimethyl *N*-cyanodithioimidocarbonate (10 mmol, 1.46 g) in ethanol (20 ml). Triethylamine (30 mmol, 3.03 g) was added. The reaction mixture was stirred overnight at room temperature. Concentrated hydrochloric acid was added; the acidified mixture for heated for an hour. The mixture was poured into ice water; the solid that formed was collected and recrystallized from ethanol to give colorless crystals of 2-(methylsulfanyl)-[1,2,4]triazolo[1,5-*a*]quinazolin-5-one. The procedure was that reported earlier (Al-Salahi & Geffken, 2011).

To the boiling mixture of 2-methylsulfanyl-[1,2,4]triazolo[1,5-*a*]quinazolin-5-one (1 mmol, 0.23 g) in glacial acetic acid (5 ml) was added hydrogen peroxide. Colorless crystals of the oxidized product were obtained when the solution was allowed to cool.

### Refinement

All H-atom were located in a difference Fourier map. Carbon-bound H-atoms were placed in calculated positions [C–H 0.93 to 0.96 Å, *U*_iso_(H) 1.2–1.5*U*_eq_(C)] and were included in the refinement in the riding model approximation.

The amino H-atom was refined isotropically with a distance restraint of N–H 0.88±0.01 Å.

### Crystal data

**Table:**

C_10_H_8_N_4_O_3_S	*F*(000) = 272
*M**_r_* = 264.26	*D*_x_ = 1.591 Mg m^−^^3^
Monoclinic, *P*2_1_	Cu *K*α radiation, λ = 1.54184 Å
Hall symbol: P 2yb	Cell parameters from 6133 reflections
*a* = 9.6216 (2) Å	θ = 3.8–76.5°
*b* = 4.9206 (1) Å	µ = 2.71 mm^−^^1^
*c* = 12.1623 (3) Å	*T* = 294 K
β = 106.628 (2)°	Plate, colorless
*V* = 551.73 (2) Å^3^	0.20 × 0.10 × 0.02 mm
*Z* = 2	

### Data collection

**Table:**

Agilent SuperNova Dual diffractometer with an Atlas detector	2304 independent reflections
Radiation source: SuperNova (Cu) X-ray Source	2237 reflections with *I* > 2σ(*I*)
Mirror monochromator	*R*_int_ = 0.025
Detector resolution: 10.4041 pixels mm^-1^	θ_max_ = 76.7°, θ_min_ = 3.8°
ω scan	*h* = −12→12
Absorption correction: multi-scan (*CrysAlis PRO*; Agilent, 2012)	*k* = −6→6
*T*_min_ = 0.613, *T*_max_ = 0.948	*l* = −14→15
9640 measured reflections	

### Refinement

**Table:**

Refinement on *F*^2^	Secondary atom site location: difference Fourier map
Least-squares matrix: full	Hydrogen site location: inferred from neighbouring sites
*R*[*F*^2^ > 2σ(*F*^2^)] = 0.030	H atoms treated by a mixture of independent and constrained refinement
*wR*(*F*^2^) = 0.082	*w* = 1/[σ^2^(*F*_o_^2^) + (0.0569*P*)^2^ + 0.056*P*] where *P* = (*F*_o_^2^ + 2*F*_c_^2^)/3
*S* = 1.04	(Δ/σ)_max_ = 0.001
2304 reflections	Δρ_max_ = 0.15 e Å^−^^3^
168 parameters	Δρ_min_ = −0.26 e Å^−^^3^
1 restraint	Absolute structure: Flack (1983), 1007 Friedel pairs
Primary atom site location: structure-invariant direct methods	Flack parameter: 0.00 (2)

### Fractional atomic coordinates and isotropic or equivalent isotropic displacement parameters (Å^2^)

**Table:**

	*x*	*y*	*z*	*U*_iso_*/*U*_eq_	
S1	0.24294 (4)	0.00109 (9)	0.18198 (3)	0.04202 (13)	
O1	0.8628 (2)	0.7880 (6)	0.13678 (16)	0.0903 (8)	
O2	0.24690 (17)	−0.1892 (3)	0.09276 (13)	0.0566 (4)	
O3	0.23731 (18)	−0.0963 (4)	0.29138 (14)	0.0645 (4)	
N1	0.67906 (18)	0.4984 (5)	0.12477 (13)	0.0542 (4)	
H1	0.677 (3)	0.451 (7)	0.0548 (13)	0.086 (9)*	
N2	0.56362 (13)	0.4813 (4)	0.27059 (11)	0.0354 (3)	
N3	0.45055 (15)	0.3499 (3)	0.29552 (13)	0.0392 (3)	
N4	0.47068 (16)	0.2190 (4)	0.12045 (12)	0.0436 (4)	
C1	0.7743 (2)	0.6953 (5)	0.18009 (17)	0.0528 (5)	
C2	0.75983 (17)	0.7852 (4)	0.29235 (15)	0.0396 (4)	
C3	0.85191 (19)	0.9855 (5)	0.35516 (16)	0.0478 (4)	
H3	0.9222	1.0634	0.3262	0.057*	
C4	0.8387 (2)	1.0679 (4)	0.45993 (18)	0.0536 (5)	
H4	0.8999	1.2021	0.5012	0.064*	
C5	0.7349 (2)	0.9521 (5)	0.50437 (17)	0.0534 (5)	
H5	0.7273	1.0095	0.5753	0.064*	
C6	0.6429 (2)	0.7530 (5)	0.44484 (16)	0.0472 (4)	
H6	0.5743	0.6733	0.4752	0.057*	
C7	0.65534 (17)	0.6748 (3)	0.33865 (14)	0.0356 (3)	
C8	0.57227 (18)	0.3991 (4)	0.16704 (14)	0.0389 (4)	
C9	0.40156 (18)	0.2013 (4)	0.20289 (14)	0.0382 (4)	
C10	0.1035 (2)	0.2349 (4)	0.12929 (18)	0.0500 (5)	
H10A	0.0116	0.1442	0.1142	0.075*	
H10B	0.1129	0.3136	0.0596	0.075*	
H10C	0.1091	0.3755	0.1851	0.075*	

### Atomic displacement parameters (Å^2^)

**Table:**

	*U*^11^	*U*^22^	*U*^33^	*U*^12^	*U*^13^	*U*^23^
S1	0.0482 (2)	0.0365 (2)	0.0405 (2)	−0.01197 (18)	0.01137 (15)	0.00488 (17)
O1	0.0929 (13)	0.131 (2)	0.0609 (10)	−0.0698 (14)	0.0448 (10)	−0.0294 (12)
O2	0.0684 (9)	0.0428 (8)	0.0593 (9)	−0.0175 (7)	0.0197 (7)	−0.0074 (7)
O3	0.0741 (10)	0.0654 (10)	0.0541 (8)	−0.0176 (8)	0.0183 (7)	0.0194 (7)
N1	0.0573 (9)	0.0762 (11)	0.0343 (7)	−0.0280 (10)	0.0216 (6)	−0.0137 (9)
N2	0.0342 (6)	0.0410 (7)	0.0312 (6)	−0.0058 (6)	0.0100 (5)	−0.0011 (6)
N3	0.0387 (7)	0.0435 (8)	0.0372 (7)	−0.0075 (6)	0.0138 (6)	−0.0003 (6)
N4	0.0456 (8)	0.0505 (9)	0.0342 (7)	−0.0125 (7)	0.0107 (6)	−0.0036 (6)
C1	0.0521 (10)	0.0692 (14)	0.0385 (9)	−0.0229 (10)	0.0150 (8)	−0.0051 (9)
C2	0.0359 (8)	0.0446 (9)	0.0360 (8)	−0.0031 (7)	0.0064 (6)	0.0006 (7)
C3	0.0431 (8)	0.0498 (10)	0.0471 (9)	−0.0101 (9)	0.0073 (7)	−0.0003 (10)
C4	0.0515 (11)	0.0499 (13)	0.0516 (11)	−0.0093 (8)	0.0022 (9)	−0.0136 (8)
C5	0.0555 (10)	0.0585 (15)	0.0452 (9)	−0.0048 (10)	0.0126 (8)	−0.0181 (9)
C6	0.0476 (9)	0.0554 (12)	0.0412 (9)	−0.0052 (9)	0.0169 (7)	−0.0098 (9)
C7	0.0342 (7)	0.0369 (8)	0.0332 (7)	0.0004 (6)	0.0055 (6)	−0.0014 (6)
C8	0.0389 (8)	0.0471 (9)	0.0296 (8)	−0.0095 (7)	0.0079 (6)	−0.0017 (7)
C9	0.0392 (8)	0.0380 (8)	0.0360 (8)	−0.0059 (7)	0.0084 (6)	0.0027 (7)
C10	0.0407 (9)	0.0498 (11)	0.0585 (11)	−0.0090 (8)	0.0127 (8)	0.0067 (9)

### Geometric parameters (Å, º)

**Table:**

S1—O3	1.4296 (16)	C1—C2	1.479 (3)
S1—O2	1.4421 (16)	C2—C7	1.395 (2)
S1—C10	1.744 (2)	C2—C3	1.397 (3)
S1—C9	1.7726 (17)	C3—C4	1.377 (3)
O1—C1	1.211 (2)	C3—H3	0.9300
N1—C8	1.364 (2)	C4—C5	1.387 (3)
N1—C1	1.370 (3)	C4—H4	0.9300
N1—H1	0.877 (10)	C5—C6	1.379 (3)
N2—C8	1.348 (2)	C5—H5	0.9300
N2—N3	1.3718 (19)	C6—C7	1.385 (2)
N2—C7	1.397 (2)	C6—H6	0.9300
N3—C9	1.312 (2)	C10—H10A	0.9600
N4—C8	1.321 (2)	C10—H10B	0.9600
N4—C9	1.355 (2)	C10—H10C	0.9600
			
O3—S1—O2	119.93 (11)	C3—C4—H4	119.8
O3—S1—C10	109.44 (11)	C5—C4—H4	119.8
O2—S1—C10	109.57 (10)	C6—C5—C4	120.88 (19)
O3—S1—C9	108.24 (9)	C6—C5—H5	119.6
O2—S1—C9	105.18 (9)	C4—C5—H5	119.6
C10—S1—C9	103.09 (9)	C5—C6—C7	118.24 (18)
C8—N1—C1	122.54 (16)	C5—C6—H6	120.9
C8—N1—H1	117 (2)	C7—C6—H6	120.9
C1—N1—H1	120 (2)	C6—C7—C2	122.18 (17)
C8—N2—N3	109.28 (14)	C6—C7—N2	122.22 (16)
C8—N2—C7	124.09 (14)	C2—C7—N2	115.60 (15)
N3—N2—C7	126.61 (13)	N4—C8—N2	111.47 (16)
C9—N3—N2	100.69 (13)	N4—C8—N1	128.56 (17)
C8—N4—C9	100.67 (15)	N2—C8—N1	119.95 (15)
O1—C1—N1	120.47 (19)	N3—C9—N4	117.88 (15)
O1—C1—C2	123.5 (2)	N3—C9—S1	121.05 (13)
N1—C1—C2	116.01 (16)	N4—C9—S1	120.94 (13)
C7—C2—C3	118.12 (18)	S1—C10—H10A	109.5
C7—C2—C1	121.66 (16)	S1—C10—H10B	109.5
C3—C2—C1	120.22 (17)	H10A—C10—H10B	109.5
C4—C3—C2	120.10 (18)	S1—C10—H10C	109.5
C4—C3—H3	119.9	H10A—C10—H10C	109.5
C2—C3—H3	119.9	H10B—C10—H10C	109.5
C3—C4—C5	120.47 (18)		
			
C8—N2—N3—C9	−0.34 (19)	C8—N2—C7—C2	0.5 (2)
C7—N2—N3—C9	178.37 (16)	N3—N2—C7—C2	−178.05 (17)
C8—N1—C1—O1	−176.2 (3)	C9—N4—C8—N2	0.2 (2)
C8—N1—C1—C2	3.1 (3)	C9—N4—C8—N1	178.8 (2)
O1—C1—C2—C7	179.4 (3)	N3—N2—C8—N4	0.1 (2)
N1—C1—C2—C7	0.1 (3)	C7—N2—C8—N4	−178.68 (16)
O1—C1—C2—C3	−0.6 (4)	N3—N2—C8—N1	−178.63 (19)
N1—C1—C2—C3	−179.9 (2)	C7—N2—C8—N1	2.6 (3)
C7—C2—C3—C4	0.4 (3)	C1—N1—C8—N4	177.0 (2)
C1—C2—C3—C4	−179.7 (2)	C1—N1—C8—N2	−4.5 (3)
C2—C3—C4—C5	0.4 (3)	N2—N3—C9—N4	0.5 (2)
C3—C4—C5—C6	−0.1 (3)	N2—N3—C9—S1	−175.38 (13)
C4—C5—C6—C7	−1.0 (3)	C8—N4—C9—N3	−0.5 (2)
C5—C6—C7—C2	1.8 (3)	C8—N4—C9—S1	175.42 (14)
C5—C6—C7—N2	−177.84 (18)	O3—S1—C9—N3	−34.09 (18)
C3—C2—C7—C6	−1.5 (3)	O2—S1—C9—N3	−163.43 (15)
C1—C2—C7—C6	178.53 (19)	C10—S1—C9—N3	81.78 (17)
C3—C2—C7—N2	178.19 (17)	O3—S1—C9—N4	150.12 (17)
C1—C2—C7—N2	−1.8 (3)	O2—S1—C9—N4	20.79 (18)
C8—N2—C7—C6	−179.84 (18)	C10—S1—C9—N4	−94.00 (18)
N3—N2—C7—C6	1.6 (3)		

### Hydrogen-bond geometry (Å, º)

**Table:**

*D*—H···*A*	*D*—H	H···*A*	*D*···*A*	*D*—H···*A*
N1—H1···O2^i^	0.88 (1)	2.23 (1)	3.072 (2)	160 (3)

Symmetry code: (i) −*x*+1, *y*+1/2, −*z*.
